# Design, Fabrication, and Characterization of a Piezoelectric Micromachined Ultrasonic Transducer with a Suspended Cantilever Beam-like Structure with Enhanced SPL for Air Detection Applications

**DOI:** 10.3390/mi16111280

**Published:** 2025-11-13

**Authors:** Yanyuan Ba, Yiming Li, Yuanhang Zhou

**Affiliations:** School of Electronics and Electrical Engineering, Henan Normal University, Xinxiang 453000, China; 1929324023@stu.htu.edu.cn (Y.L.); 2322283017@stu.htu.edu.cn (Y.Z.)

**Keywords:** piezoelectric micromachined ultrasonic transducers (PMUTs), microelectromechanical systems (MEMS), air-coupled ultrasonic detection, cantilever beam structure, boundary-suspended diaphragm, sound pressure level

## Abstract

Air-coupled ultrasonic detection demands high transmission performance from piezoelectric micromachined ultrasonic transducers (PMUTs). However, existing microelectromechanical system (MEMS)-based PMUTs deliver limited output, which compromises measurement accuracy and constrains further development. This work proposes a novel PMUT design with a cantilevered, boundary-suspended diaphragm that relieves residual stress, relaxes edge constraints, increases the mechanical degrees of freedom, and enables larger vibration amplitudes. Additionally, this work develops an accurate air-coupling model to predict device performance and a streamlined micro-nanofabrication process for device realization. Experimental results show that under a 1 Vpp (−5 Voffset) drive, the device achieves a peak acoustic pressure of 4.004 Pa at 69.3 kHz, measured at 10 cm distance in air, corresponding to a maximum sound pressure level of 106.02 dB (re 2 × 10^−5^ Pa). Compared to a traditional PMUT at 98.45 dB, this represents a 7.57 dB improvement and, to our knowledge, the highest reported sound pressure level at 10 cm for a single PMUT operating near 70 kHz under a 1 Vpp excitation. These results validate the significant enhancement in transmission performance achieved by the proposed topological structure, offering a solution to overcome the common bottleneck of insufficient output in PMUTs, and indicate strong potential for broader air-coupled sensing applications.

## 1. Introduction

With the rapid development of emerging applications such as intelligent robots and unmanned aerial vehicles (UAVs), mid- to short-range detection technology has increasingly become a key technology for precise positioning and target identification. Ultrasonic detection technology is not limited by factors such as object color, transparency, and ambient noise, offering significant advantages over infrared detection, radar detection, and other technologies [1,2,3,4,5,6]. With the development of microelectromechanical system (MEMS) technology, micromachined ultrasonic transducers (MUTs), characterized by smaller size, easier integration, and lower power consumption, have replaced traditional piezoelectric ceramic transducers as the core components of ultrasonic detection. Since CMUTs typically require complex drive circuit designs and exhibit undesirable nonlinear responses, PMUTs, featuring simpler drive schemes and superior controllability, have emerged as a more promising alternative for ultrasonic detection applications [7,8,9,10]. Air-coupled ultrasonic detection imposes stringent requirements on the transmission performance of transducers. Given the attenuation and loss of sound wave signals over a certain distance in the air, the stronger the transmitted signal, the smaller the attenuation and interference of the echo signal, leading to higher detection accuracy. However, although current PMUTs demonstrate excellent sensitivity in receiving modes, their transmission performance remains severely constrained by structural limitations and residual stresses introduced during fabrication, falling well below desired levels. This significantly limits the accuracy of airborne ultrasonic detection [11,12]. Extensive research has been devoted to enhancing the transmission sensitivity of individual PMUTs. From a material perspective, the use of scandium-doped aluminum nitride (AlScN) as the piezoelectric material has proven effective in improving the transmission performance of PMUTs [13,14]. Yao S et al. proposed a single Sc_0.2_Al_0.8_N PMUT based on a bent suspended membrane, equipped with a horn-like gain device, achieving a high sensing range of 11.2 m [15]. However, the high cost of AlScN and the additional complexities it introduces into fabrication processes pose significant challenges for large-scale implementation in ultrasonic sensor arrays. From a structural design perspective, modulating the stiffness of the diaphragm to alter the operating mode of PMUTs has become a primary focus of research. Tao W et al. introduced three concentric circular etched holes at the center of the PMUT diaphragm, transforming its vibration mode from Gaussian-like to piston-like. The piston-mode PMUT demonstrated a transmission sound pressure level 5.3 dB higher than that of traditional PMUTs [16]. Xu Ying Chen et al. proposed a PMUT structure in which a ring of V-shaped spring holes was etched onto the diaphragm surface, enabling the PMUT to operate in piston mode. The output sound pressure of this piston-mode PMUT was enhanced by 23 dB compared with PMUTs without the spring design [17]. Although these designs improve transmission performance to a certain extent, the densely etched structures complicate fabrication and increase the mechanical fragility of vibrating components, thereby exacerbating sound leakage. Moreover, residual stress cannot be completely confined to the intended softened regions of the diaphragm; as a result, only a piston-like vibrational mode is achieved, and transmission sensitivity sees no significant improvement. In light of these limitations, cantilever beam architectures, which permit larger deflections and inherently relieve internal stress, have been explored as an alternative approach to enhance PMUT performance. Recently, Yan Wang et al. reported a PDMS-sealed PMUT based on a cantilever beam structure, where PDMS acts as a soft interconnecting spring between adjacent cantilever beams, effectively suppressing asynchronous vibrations in the PMUT device. In a pulse-echo rangefinding experiment, driven by a 40 Vpp sinusoidal signal at a resonant frequency of 69.2 kHz, the PMUT achieved a maximum detection range of 3.8 m [18]. However, PDMS is highly sensitive to environmental light, which accelerates material aging and leads to degradation of the cooperative behavior among cantilever beams. This deterioration significantly reduces the output performance of the PMUT and restricts its applicability in practical scenarios. To address these limitations, this work proposes a novel structural design for the PMUT, innovatively introducing micro-slit and trapezoidal hole etching structures. These designs not only ensure the diaphragm strength but also enable the cantilever beam structure and quasi-suspended membrane structure to effectively release stress, while avoiding the out-of-phase operation mode of the cantilever beams and the associated sound leakage effects. The designed quasi-suspended membrane structure significantly enhances the vibration amplitude of the PMUT, thereby improving its transmission performance.

## 2. Materials and Methods

### 2.1. Device Design

Traditional PMUT structures typically adopt a fixed boundary design, where the diaphragm boundaries are strictly constrained by surrounding structures. This design process is simple and well-established; however, the diaphragm’s freedom of movement is significantly restricted due to the fixed boundaries. The boundary constraints lead to the accumulation of localized stress and stiffness, which restrict the vibration amplitude of the diaphragm, thereby affecting the transmission performance and efficiency of the PMUT [19,20].

**Figure 1:**
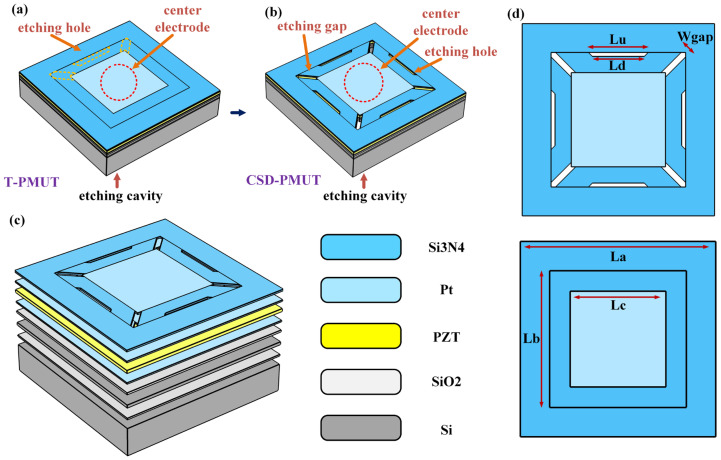
Geometric design and structural schematic of the CSD-PMUT. (**a**) Schematic of the traditional PMUT design, highlighting the structural modifications of the CSD-PMUT relative to the traditional PMUT. (**b**) Schematic of the CSD-PMUT design, showing the trapezoidal etched holes and micro-slit structure. (**c**) Exploded schematic of the proposed CSD-PMUT structure. (**d**) Geometric parameter design diagram of the PMUT diaphragm.

Figure 1a shows a schematic of the traditional PMUT (T-PMUT). To overcome the limitations in vibration of the traditional PMUT, the yellow dashed portion in Figure 1a represents the proposed innovative etching topology design. Figure 1b presents a schematic of the proposed new design PMUT with cantilever beam suspension diaphragm (CSD-PMUT). The CSD-PMUT introduces trapezoidal etched holes and diagonal micro-slit structures at the diaphragm boundary. The design of the trapezoidal holes effectively releases stress, reduces the constraint and mechanical stiffness at the diaphragm boundary, and enhances the freedom of movement at the boundary, forming a quasi-suspended diaphragm. The introduction of the micro-slit structure results in a diaphragm resembling a cantilever beam, with the unetched square platform at the center serving as a connecting structure for the cantilever beams, allowing them to move in unison. This enables the diaphragm to achieve larger vibration amplitudes during motion, thereby enhancing the transmission performance of the PMUT. Figure 1c shows the exploded schematic of the CSD-PMUT structure. The PMUT includes an SOI structure, a SiO_2_ oxide layer, upper and lower platinum metal electrodes, a PZT piezoelectric layer, and a Si_3_N_4_ protective layer. The thickness of each structural layer is listed in Table 1. The geometric parameter design diagram of the diaphragm for both the CSD-PMUT and T-PMUT is shown in Figure 1d, detailing the chip size, central platform, micro-slits, trapezoidal holes, and other key geometric features, including their dimensions and layout. The specific geometric parameters of the PMUT are listed in Table 2. The overall dimensions of the PMUT are approximately 2.5 × 2.5 mm^2^, with a diaphragm area of 1.7 × 1.7 mm^2^, achieving a 68% top electrode coverage, thus maximizing the excitation efficiency [21]. To simplify the nomenclature, the new PMUT design proposed in this study is referred to as CSD-PMUT, while the traditional non-etched structure PMUT is referred to as T-PMUT.

### 2.2. Device Concept

Based on the inverse piezoelectric effect, the region of the piezoelectric thin film covered by the top electrode in a PMUT structure undergoes inward electromechanical strain. The stress gradient along the film thickness direction generates a bending moment about the neutral axis of the film. According to the piezoelectric constitutive equations, the stress *d_p_* produced by the piezoelectric layer and the corresponding bending moment *M_p_* exerted by this layer on the neutral axis are defined as follows [22,23]:

(1) $$\delta_{p}=Y_{p}\cdot d_{31}\cdot E_{z}$$

(2) $$M_{p}=Y_{p}\cdot d_{31}\cdot Z_{p}\cdot V_{p}$$

where *Y_P_* is the Young’s modulus of PZT, *d*_31_ is the piezoelectric constant, *E_Z_* is the electric field strength, *Z_p_* is the distance from the middle of the piezoelectric layer to the neutral axis of the diaphragm, and *V_p_* is the voltage applied to the PMUT piezoelectric layer. The transmission performance of a PMUT under excitation can be evaluated by its electromechanical efficiency, defined as the ratio of the output mechanical energy to the input electrical energy. For a traditional PMUT architecture, the input electrical energy can be expressed as follows [24]:

(3) $$E_{e}=\frac{1}{2}\left(\frac{0.68A_{P}\varepsilon_{P}}{h_{p}}\right){V_{p}}^{2}=\frac{17}{50}\left(\frac{a^{2}\varepsilon_{P}}{hp}\right){V_{p}}^{2}$$

Here, 0.68 denotes the electrode-coverage index that yields optimal excitation of the PMUT, *A_p_* is the diaphragm area, *a* is the side length of the square diaphragm, *h_p_* is the thickness of the piezoelectric layer, and *e_p_* is the permittivity. Accordingly, the output mechanical energy of a traditional PMUT is given by the following [25]:

(4) $$E_{m}=\frac{1}{2}k_{mC}{X_{C}}^{2}$$

(5) $$X_{C}=\frac{M_{p}}{k_{mC}}$$

Here, *k_mC_* denotes the effective mechanical stiffness at the diaphragm center of the PMUT, and *X_C_* is the center displacement. According to the electromechanical relations, the electromechanical efficiency of a traditional PMUT is given by the following [25]:

(6) $$\eta_{e}=\frac{E_{m}}{E_{e}}=\frac{25}{17}\left(\frac{h_{p}k_{mc}}{a^{2}\varepsilon_{p}}\right){\left(\frac{X_{C}}{V_{p}}\right)}^{2}$$

Introducing micro-slits and etched apertures into the PMUT diaphragm relaxes the boundary constraints and lowers the film stiffness; the resulting quasi-cantilever configuration substantially increases the vibrational displacement and effectively relieves residual stress. For a PMUT incorporating such etching features, if the stiffness reduction induced by perforation is represented by a coefficient ***w*** (0 < *w* < 1), the corrected mechanical stiffness of the diaphragm can be defined as follows:

(7) $${k_{mc}}^{*}=\omega \cdot k_{mc}$$

Accordingly, the output mechanical energy of the PMUT with the modified etched architecture can be expressed as follows:

(8) $${E_{m}}^{*}=\frac{1}{2}\frac{{M_{p}}^{2}}{{\left(1-\omega \right)}^{2}k_{mC}}$$

According to Equation (5) to Equation (8), the electromechanical efficiency of the perforated PMUT with stiffness correction was calculated using the following formula:

(9) $${\eta_{e}}^{*}=\frac{{E_{m}}^{*}}{E_{e}}=\frac{1}{{\left(1-\omega \right)}^{2}}\eta_{e}$$

Consequently, the electromechanical efficiency of a PMUT incorporating etched perforations is 1/(1 − *w*)^2^ times that of a traditional, non-perforated device, indicating a pronounced improvement in efficiency enabled by the perforated architecture.

The sound pressure level (SPL) is a metric of acoustic intensity at a specific location within a medium. It is defined as the logarithmic ratio between the maximum acoustic pressure and a reference pressure in that medium, and it is reported in decibels (dB). Its expression is as follows:

(10) $$SPL=20\log_{10}\left(\frac{P}{P_{ref}}\right)$$

Here, *P* denotes the acoustic pressure at the measurement point within the medium, and *P_ref_* is the reference acoustic pressure in that medium, typically 20 × 10^−6^  Pa in air. The *SPL* maps the pressure amplitude to a comparable decibel (dB) scale, thereby facilitating the comparative assessment of PMUT transmission performance.

### 2.3. Three-Dimensional Finite Element Simulation Research

The multiphysics coupling of solid mechanics, electrostatic fields, and pressure acoustics in COMSOL 6.3 provides a powerful simulation platform for modeling the frequency modes, vibrations, and acoustic performance of the PMUT [26,27]. This work employs COMSOL Multiphysics 6.3 for a comprehensive simulation study of the CSD-PMUT and T-PMUT. First, a 3D model of the PMUT was constructed in COMSOL based on its geometry and structure. Then, the material properties and boundary conditions for each region were refined. The material properties used in the simulation are detailed in Table 3. In the solid mechanics physics field, fixed constraints were applied to the bottom surface of the SOI substrate, while in the electrostatic field, terminal voltage and grounding conditions were set on the upper and lower surfaces of the PZT to effectively excite the PMUT. To simulate the acoustic characteristics of the PMUT, a hemisphere air domain with a radius of six wavelengths was added to the surface of the PMUT in the pressure acoustics physics field. A perfect matching layer with a thickness of half the wavelength was applied at the boundary to model the infinite propagation of sound waves and prevent reflection interference within the computational domain. An external field calculation boundary was also set up to simulate the sound pressure at any position in the external field.

To ensure the accuracy and mesh independence of the simulation results, the mesh for the air domain was set to one-sixth of the wavelength. Additionally, a boundary layer mesh with a thickness of one-hundredth of the wavelength was added at the interface between the air domain and the perfect matching layer, ensuring the accuracy and stability of the simulation results.

### 2.4. Device Manufacturing

**Figure 2:**
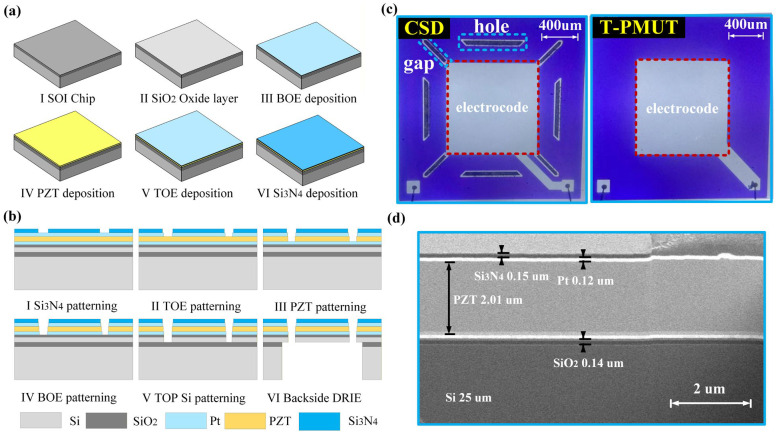
Micro-nano electronic process flowchart of the PMUT and microscope inspection image of the fabricated PMUT sample. (**a**). Thin film deposition process flowchart: I. SOI wafer, II. SiO_2_ oxide layer, III. bottom electrode deposition, IV. piezoelectric layer deposition, V. top electrode deposition, and VI. protective layer deposition. (**b**). Etching process flowchart: I. patterning of the protective layer, II. patterning of the top electrode, III. patterning of the PZT, IV. patterning of the bottom electrode, V. patterning of the top silicon, VI. back-silicon DRIE etching and cavity release. (**c**). Microscope inspection images of the diaphragms of CSD-PMUT and T-PMUT. (**d**). Cross-sectional scanning electron microscopy (SEM) image of the PMUT, revealing the well-defined piezoelectric layer deposition structure.

The device fabrication is based on a simple and low-cost micro-nano electronic manufacturing process. The micro-nano fabrication process flowchart of the PMUT and the microscope inspection image of the fabricated PMUT sample are shown in Figure 2. Figure 2a shows the device thin film deposition process flowchart. I: A commercial SOI wafer with 25 μm silicon, a 1 μm SiO_2_ buried oxide layer, and 410 μm silicon is used as the substrate. II: The SiO_2_ oxide layer is grown using a thermal oxidation method, with a thickness controlled around 150 nm, forming the necessary electrical isolation layer. After the SiO_2_ oxide layer growth is complete, the electrode deposition stage begins. III: A 100 nm thick bottom electrode Pt layer is deposited via sputtering, with the electrode layer exhibiting good uniformity and adhesion. IV: The PZT material is uniformly coated using the sol–gel method, followed by high-temperature annealing to form a dense piezoelectric film. After the deposition of the piezoelectric layer, the top electrode Pt layer is also deposited using sputtering deposition technology. VI: A 150 nm thick Si_3_N_4_ protective layer is deposited on the surface using PECVD to prevent damage from the external environment and enhance the robustness and lifespan of the PMUT [7,28].

After completing the thin film deposition, the device undergoes photolithography and etching processes, as shown in Figure 2b. I. Photoresist is applied to the protective layer, and ultraviolet lithography is used to transfer the designed pattern onto the photoresist. After development, the unexposed photoresist is removed, exposing the areas of Si_3_N_4_ to be etched. A dry etching process using fluorine-based gases is employed to remove the unprotected Si_3_N_4_ areas. II. Patterning of the top electrode Pt layer is performed using a photolithography process similar to Step I to define the pattern of the top electrode. Argon ion beam etching is used to remove the unprotected Pt layer. III. Patterning of the PZT layer is performed by defining its pattern using a photolithography process similar to Step I. A mixed HCl/HF solution is used for selective etching of the PZT, with the bottom electrode Pt layer serving as the etch stop layer. IV. Patterning of the bottom electrode Pt layer is carried out similarly to Step II, ensuring that the bottom electrode is accurately aligned with the PZT pattern above it. V. Patterning of the top silicon is performed by photolithography to define the areas to be etched. DRIE is then used to deep-etch the top silicon layer down to the buried oxide layer, forming the anchor areas and side profiles of the movable parts of the suspended structure. Finally, back-silicon DRIE etching and cavity release are performed, as shown in VI: First, photolithography is applied to the back of the wafer to align the cavity pattern with the front device region. Deep reactive ion etching (DRIE) technology is used to perform high-aspect-ratio deep etching of the silicon substrate, automatically stopping at the buried oxide layer. A hydrofluoric acid solution is used to selectively remove the exposed silica buried layer, completely releasing the diaphragm structure and forming the micro-cavity. An optical micrograph of the PMUT prototype after laser wafer dicing and wire bonding of the electrodes is shown in Figure 2c. Compared to the diaphragm of the T-PMUT, the diagonal micro-slit, four trapezoidal apertures, and four cantilever beam structures of the CSD-PMUT are clearly visible. The blue Si_3_N_4_ protective layer on the PMUT surface and the pale central top electrode are apparent, and the bottom electrode region is connected by wire bonds. The cross-sectional scanning electron microscope (SEM) image of the PMUT shown in Figure 2d demonstrates the standard piezoelectric sandwich structure of the PMUT. The excellent deposition condition provides fundamental process-level support for the precise characterization of PMUT performance.

## 3. Results

### 3.1. Impedance and Modal Analysis

**Figure 3:**
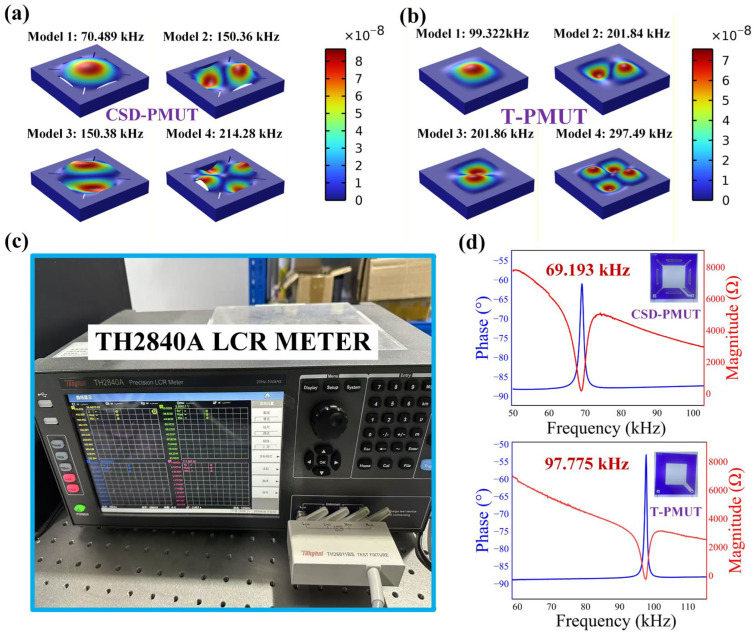
Three-dimensional finite element simulation modes and impedance test results of different PMUTs. (**a**). The first four modal shapes of the CSD-PMUT. (**b**). The first four modal shapes of the T-PMUT. (**c**). The impedance analyzer used in the impedance testing experiment. (**d**). Impedance and phase diagrams of CSD-PMUT and T-PMUT.

In the 3D finite element simulation experiment, the first four modes of the CSD-PMUT and T-PMUT were obtained using COMSOL’s piezoelectric coupling multiphysics and characteristic frequency solvers. The modal diagram of CSD-PMUT is shown in Figure 3a. The first mode is a Gaussian mode with a resonant frequency of 70.489 kHz, the second and third modes have resonant frequencies of 150.36 kHz and 150.38 kHz, respectively, and the fourth mode has a resonant frequency of 214.28 kHz. The modal diagram of T-PMUT is shown in Figure 3b. The first resonant frequency is 99.322 kHz, corresponding to a typical Gaussian mode, with the second and third modes having resonant frequencies of 201.84 kHz and 201.86 kHz, respectively, and the fourth mode resonant frequency being 297.49 kHz. Considering that the energy and vibration of the first mode are more concentrated, and that other modes exhibit some degree of mode coupling, which makes them difficult to excite under actual test conditions, the first mode is chosen as the operating mode. From the CSD-PMUT first mode diagram, it can be seen that the design of the etched holes around the diaphragm increases the boundary degrees of freedom of the diaphragm. Figure 3c shows the TH2840A model LCR tester. After connecting the PMUT to the instrument and setting the frequency sweep range, the actual impedance and other data of the PMUT were obtained. The PMUT’s measured impedance and related parameters were obtained from multiple test sets conducted under identical experimental conditions. The test data for impedance and phase of the PMUT are shown in Figure 3d. It can be observed that the actual first resonant frequency of CSD-PMUT is 69.193 kHz, which differs by 1.296 kHz from the simulated value of 70.489 kHz. The actual first resonant frequency of T-PMUT is 97.775 kHz, which differs by 1.547 kHz from the simulated value of 99.322 kHz. The simulation and actual values match well, with an error within 1.87%. The error may arise from incomplete etching in the manufacturing process and differences in material properties. The first resonant frequency of CSD-PMUT is lower than that of T-PMUT, which is due to the mass and inertia loss introduced by the etched structure in CSD-PMUT.

### 3.2. Resonant Displacement

**Figure 4:**
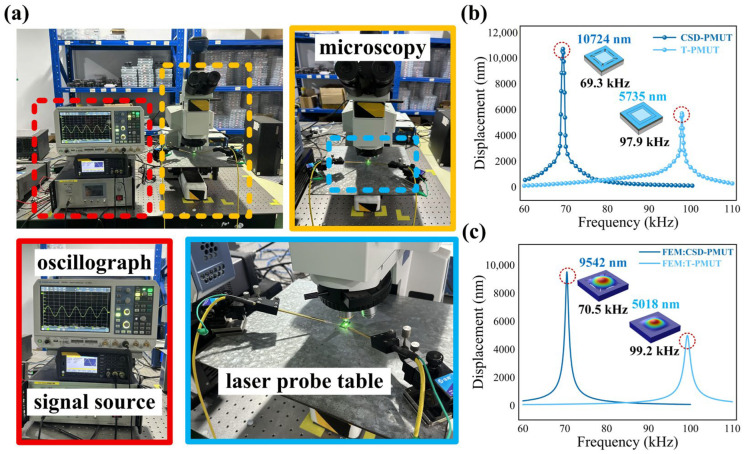
Schematic of the LDV testing experiment platform and the simulation and test results of the PMUT resonant displacement. (**a**). Setup of the oscilloscope, signal source, microscope, and laser Doppler probe station. (**b**). Frequency and resonant displacement point-line diagram of different PMUTs from LDV experimental testing. (**c**). Frequency and resonant displacement line chart of different PMUTs obtained from simulation experiments.

Resonant displacement is a key indicator of PMUT transmission performance. In this experiment, resonant displacement of the PMUT was tested under normal air conditions using the LDV laser Doppler platform. Figure 4a shows the instrumentation setup of the resonant displacement testing platform, where the oscilloscope and signal source are used to output voltage excitation at a specific amplitude and frequency, and the microscope and LDV laser Doppler probe station are used for device alignment and resonant displacement measurement at the center of the device. In the experiment, a 1 Vpp (−5 Voffset) sinusoidal voltage was applied to the PMUT, and the LDV laser was focused on the maximum displacement point at the center of the PMUT diaphragm. Fine testing was performed at five points above and below the resonant frequency obtained from the impedance test with a step size of 0.1 kHz, while other frequencies were tested with a step size of 1 kHz. Resonant displacement data acquired from multiple replicate tests conducted under identical conditions are shown in Figure 4b. The resonant peak for CSD-PMUT appears at 69.3 kHz, with a corresponding maximum resonant displacement of 10,724 nm. The maximum resonant displacement for T-PMUT is 5735 nm, occurring at 97.9 kHz. The simulation results of resonant displacement under the same excitation conditions are shown in Figure 4c. The resonant peak for CSD-PMUT appears at 70.5 kHz, with a maximum resonant displacement of 9542 nm. The maximum resonant displacement for T-PMUT is 5018 nm, occurring at 99.2 kHz. The good fit between the simulation and actual test values demonstrates the accuracy of the designed simulation model. The resonant displacement of CSD-PMUT is 4989 nm higher than that of T-PMUT; this is because the etched holes and cantilever beam structure increase the diaphragm’s degrees of freedom, which amplifies the diaphragm’s vibrational displacement.

**Figure 5:**
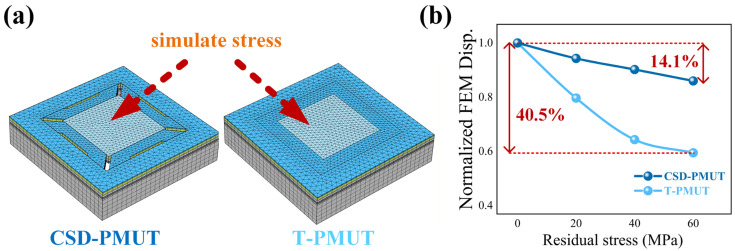
Simulation setup and results of the stress sensitivity of the PMUT. (**a**). A gradient stress was applied to the vibrating structure of the PMUT to simulate the effect of residual stress. (**b**). A point-line plot depicting the normalized simulated resonant displacement of the PMUT as a function of the residual stress.

To further investigate the efficacy of the designed structure in mitigating residual stress, a stress robustness simulation was established in COMSOL, with the model schematic illustrated in Figure 5a. The CSD-PMUT and T-PMUT served as structural controls. Residual stress was applied to the vibrating structures of both PMUTs, increasing from 0 MPa in 20 MPa increments, followed by frequency response simulations. The resultant normalized displacement of the PMUTs under the applied stress is shown in Figure 5b. It can be observed that under the influence of stress ranging from 0 to 60 MPa, the normalized output displacement of the T-PMUT degrades by 40.5%, whereas that of the CSD-PMUT exhibits a markedly smaller degradation of only 14.1%, with a more gradual rate of change. These simulation results indicate that the quasi-cantilever configuration, introduced via micro-slits and etching holes, effectively releases in-plane residual stress by weakening the boundary constraints. This mechanism substantially reduces the sensitivity of the device’s performance to process-induced residual stress. This conclusion is consistent with the resonant displacement measurements presented in Figure 4, where the CSD-PMUT achieves a larger displacement.

### 3.3. Acoustic Performance

**Figure 6:**
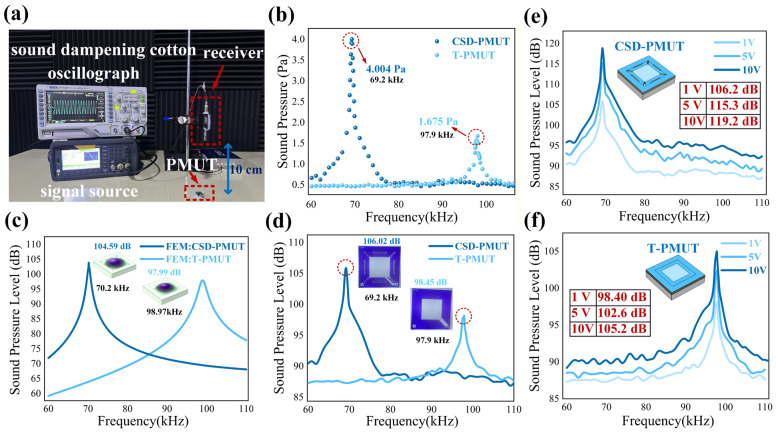
Schematic of the acoustic testing platform and the simulation and test results of the acoustic performance. (**a**). Schematic of the acoustic testing platform, including the oscilloscope, signal source, and acoustic silencing device, with the PMUT and receiving probe positioned 10 cm apart. (**b**). Transmission sound pressure test scatter plot for CSD-PMUT and T-PMUT at 10 cm in an air environment. (**c**). Simulation results of resonant frequency vs. sound pressure level for different PMUTs. (**d**). Sound pressure level test results for CSD-PMUT and T-PMUT at 10 cm in an air environment. (**e**,**f**). Sound pressure level at 10 cm versus resonant frequency of the PMUT under various driving voltages.

Sound pressure and sound pressure level can directly measure the acoustic transmission performance of the PMUT in a medium. The acoustic performance testing conditions and resonant displacement testing calibration are performed by measuring the sound pressure and sound pressure level of the device at a position 10 cm above the device under normal atmospheric conditions. The configuration of the acoustic performance testing platform is shown in Figure 6a.

The acoustic testing platform is set up in a sealed environment surrounded by soundproofing devices, which significantly reduces the interference of ambient noise on the testing. The oscilloscope and signal source are used to output voltage excitation with adjustable amplitude and frequency. To precisely and quantitatively characterize the emission performance of the proposed PMUT, a standard microphone was employed for transmitting sound pressure measurements. This measurement scheme effectively avoids errors inherent in the pulse-echo method, such as transducer response coupling and electrical crosstalk, thereby serving as a standard methodology for the quantitative assessment of transducer emission performance. The acoustic wave generated by the excited PMUT was received by a high-precision 1/4-inch standard free-field measurement microphone (SoundFree M300-4F, SoundFree LIMITED, Beijing, China), positioned 10 cm directly above the device. The microphone was connected to a dedicated preamplifier and power supply, exhibiting a flat sensitivity frequency response (±1 dB) within the range of 20 Hz to 100 kHz. The output voltage from the microphone was recorded using a digital acquisition card and subsequently converted to the corresponding sound pressure level [29]. Under a 1 Vpp (−5 Voffset) sinusoidal AC voltage excitation, the maximum acoustic pressure values measured across multiple trials under identical conditions are shown in Figure 6b. CSD-PMUT generates a sound pressure of 4.004 Pa at 69.2 kHz at a distance of 10 cm in air, while T-PMUT generates a maximum sound pressure of 1.675 Pa at 97.9 kHz. The sound pressure level simulation values obtained from the COMSOL air-coupled simulation model under the same excitation and air conditions are shown in Figure 6c. CSD-PMUT has a maximum sound pressure level of 104.59 dB at 70.2 kHz, while T-PMUT generates a sound pressure level of 97.99 dB at 98.97 kHz. The actual tested sound pressure levels are shown in Figure 6d. CSD-PMUT has a maximum sound pressure level of 106.02 dB at 69.2 kHz, while T-PMUT generates a sound pressure level of 98.45 dB at 97.9 kHz. The simulation values fit well with the actual test results. Compared to T-PMUT, CSD-PMUT’s sound pressure at 10 cm in the air increased by 2.329 Pa under 1 Vpp excitation, and the sound pressure level increased by 7.57 dB. Due to CSD-PMUT’s low-edge-constraint suspended diaphragm design, it achieves higher resonant displacement and enhanced acoustic output. This is currently the highest reported sound pressure level at 10 cm in the air for PMUTs in the 70 kHz frequency range. Figure 6e,f depict the sound pressure level at 10 cm versus the resonant frequency of the PMUTs under various driving voltages. As the driving voltage increases, the output SPL rises, albeit with a sublinear growth trend, accompanied by a moderate enhancement in bandwidth. At a driving voltage of 10 V, the CSD-PMUT achieves a high SPL of 119.2 dB at 10 cm, demonstrating excellent and stable acoustic output, while an SPL of 105.3 dB is measured for the T-PMUT. The excellent acoustic performance of CSD-PMUT in air verifies the improvement in PMUT transmission performance brought by the proposed novel topological structure.

## 4. Discussion

This work presents a novel PMUT based on a cantilever beam-like suspended membrane, with the diaphragm featuring a micro-slit and edge trapezoidal etching hole design. First, the enhancement of the PMUT transmission performance due to this design was analyzed theoretically, followed by finite element simulation to verify the performance improvement of the novel geometric topology. The prototype was then fabricated, and its performance was verified through LDV and acoustic experiments in air, successfully validating the excellent transmission performance of the new design. The developed simple micro-nano electronic process enables low-cost, large-scale manufacturing of the device. This study focuses on the performance characterization of a single PMUT chip. Its excellent acoustic performance allows for expansion into large-scale PMUT array applications and extends its potential for applications in air-based systems, such as 3D object detection [30]. The transmission performance of the PMUT developed in this work is compared with the acoustic performance of existing PMUTs, as shown in Table 4. Ref. [18] reports a PMUT operating at the same frequency as the present work; while it achieves high transmission performance per unit diaphragm area at a distance of 10 cm, the measurement was conducted under a 40 V drive. For instance, a PZT-based PMUT with a diaphragm area equivalent to that in our work is reported in Ref. [31], which achieves a sound pressure of 2.01 Pa at 10 cm under a 5 V drive at 95 kHz; this yields a lower performance compared to our device. Ref. [32] likewise describes a PMUT operating at the same frequency as this work and delivering high transmission performance per unit diaphragm area at 10 cm, but again under a 40 V drive. Ref. [33] attains a high transmission sound pressure of 5.9 Pa at 26 cm. However, it utilizes high-performance PZT with a piezoelectric coefficient enhanced by specialized processing, and the test configuration employs a 2 × 2 array. Meanwhile, Ref. [34] reports an AlN-based PMUT resonating at 80 kHz. Despite its considerably smaller emission area, this device generates a sound pressure of only 2.55 Pa at a distance of 1 cm in air under 5 V drive, alongside a relatively low resonant displacement. By contrast, the PMUT developed here delivers large displacement and high peak acoustic pressure under low-voltage excitation, while maintaining a small diaphragm area and a compact device footprint. It should be noted that this work has achieved a breakthrough by focusing on the enhancement of transmission performance of the proposed novel structure in low-frequency PMUTs for airborne detection. Future research may extend these investigations to high-frequency regimes and biological applications, thereby exploring its potential for high-resolution ultrasonic imaging. It should also be noted that the high transmission performance of our design is achieved at the expense of a narrow bandwidth, which may limit its applicability in certain scenarios [35]. Therefore, addressing this bandwidth limitation represents a key focus for future research. Potential strategies for bandwidth enhancement in subsequent designs include the implementation of a PMUT array composed of elements with slight frequency offsets, whose collective response can synthesize a broader operational bandwidth. Additionally, the modification of driving strategies offers another viable avenue for improving bandwidth.

## 5. Conclusions

This work presents a novel PMUT design based on a cantilever beam-like suspended membrane, introducing trapezoidal etched holes and micro-slit structures at the diaphragm boundary. The design of the trapezoidal holes and micro-slits effectively releases stress, reduces the constraints and mechanical stiffness at the diaphragm boundary, and increases the diaphragm’s degrees of freedom. This results in a cantilever beam-like suspended structure, with a central square platform serving as the connecting structure for the cantilever beams, allowing them to move in unison. This enables the diaphragm to achieve larger vibration amplitudes during motion, thereby enhancing the transmission performance of the PMUT. Experimental results show that under a 1 Vpp (−5 Vos) low drive voltage, the maximum resonant displacement of the CSD-PMUT is 4849 nm higher than that of the traditional PMUT. The maximum sound pressure at 10 cm in the air external field can reach 4.004 Pa, and the maximum sound pressure level reaches 106.02 dB, an increase of 7.57 dB over the traditional PMUT’s 98.45 dB. This is currently the highest reported sound pressure level for a single PMUT at 10 cm in the external field in the 70 kHz frequency range. This validates the improvement in the transmission performance of the PMUT brought about by the proposed novel topological design, providing a solution to the current bottleneck of low PMUT transmission performance and demonstrating broad prospects for expanded applications in air detection.

## Figures and Tables

**Table 1:** Summary of structural parameters.

Symbol	Value	Description
H_PZT	2 um	Thickness of piezoelectric layer
H_Si	25 um	Thickness of vibrating layer of silicon
H_BOX	1 um	Thickness of SOI buried oxygen layer
H_SUB	400 um	Thickness of SOI silicon substrate
H_Pt	0.1 um	Thickness of electrodes
H_SiO_2_	0.16 um	Thickness of the oxide layer
H_Si_3_N_4_	0.16 um	Thickness of the protective layer

**Table 2:** Summary of geometric parameters.

Symbol	Value	Description
L_a	2425 um	Side length of the PMUT
L_b	1700 um	Side length of the cavity
L_c	1190 um	Side length of the top electrode
L_u	755 um	Length of the lower base of the trapezoidal hole
L_d	655 um	Length of the upper base of the trapezoidal hole
W_Gap	20 um	Width of the micro-gap

**Table 3:** Material properties in finite element simulation.

Symbol	Description	Value	Unit
YSi	Si Young’s modulus	140	GPa
vSi	Si Poisson’s ratio	0.28	1
YSiO_2_	SiO_2_ Young’s modulus	70	GPa
YSi_3_N_4_	Si_3_N_4_ Young’s modulus	250	GPa
e_31_PZT	PZT piezoelectric coefficient	−3.24	C/m^2^
rPZT	PZT density	7750	kg/m^3^
rPt	Pt density	21,450	kg/m^3^
rSiO_2_	SiO_2_ density	2200	kg/m^3^

**Table 4:** Performance comparison of the PMUTs from the literature.

Resonant Frequency	Piezoelectric Material	Upp [V]	Diaphragm Area [mm^2^]	Displacement [um]	Max Sound Pressure [Pa]	Reference
69.3 kHz	PZT	1 + −5 Vos	1.7	10.7	4.004/10 cm/1 cell	This work
95 kHz	PZT	5	1.7	/	2.01/10 cm/1 cell	Ref. [31]
58 kHz	PZT	40	2.25	4.2	3.61/8 cm/1 cell	Ref. [32]
48 kHz	S-C-PZT	10	9	15	5.9/26 cm/4 cells	Ref. [33]
69.2 kHz	ALN	40	0.518	/	1.89/10 cm/1 cell	Ref. [18]
80 kHz	ALN	5	0.5	2.34	2.55/1 cm/1 cell	Ref. [34]

## Data Availability

The original contributions presented in this study are included in the article. Further inquiries can be directed to the corresponding author.
